# Novel metagenome-assembled genomes involved in the nitrogen cycle from a Pacific oxygen minimum zone

**DOI:** 10.1038/s43705-021-00030-2

**Published:** 2021-06-18

**Authors:** Xin Sun, Bess B. Ward

**Affiliations:** 1 Department of Geosciences, Guyot Hall, Princeton University, Princeton, NJ USA; 2 Present Address: Department of Ecology and Evolutionary Biology, Yale University, New Haven, CT USA; 3 Present Address: Microbial Sciences Institute, Yale University, West Haven, CT USA; 4 Present Address: Yale Institute for Biospheric Studies, Yale University, New Haven, CT USA

**Keywords:** Microbial ecology, Water microbiology

## Abstract

Oxygen minimum zones (OMZs) are unique marine regions where broad redox gradients stimulate biogeochemical cycles. Despite the important and unique role of OMZ microbes in these cycles, they are less characterized than microbes from the oxic ocean. Here we recovered 39 high- and medium-quality metagenome-assembled genomes (MAGs) from the Eastern Tropical South Pacific OMZ. More than half of these MAGs were not represented at the species level among 2631 MAGs from global marine datasets. OMZ MAGs were dominated by denitrifiers catalyzing nitrogen loss and especially MAGs with partial denitrification metabolism. A novel bacterial genome with nitrate-reducing potential could only be assigned to the phylum level. A Marine-Group II archaeon was found to be a versatile denitrifier, with the potential capability to respire multiple nitrogen compounds including N_2_O. The newly discovered denitrifying MAGs will improve our understanding of microbial adaptation strategies and the evolution of denitrification in the tree of life.

## Main

**Fig. 1:**
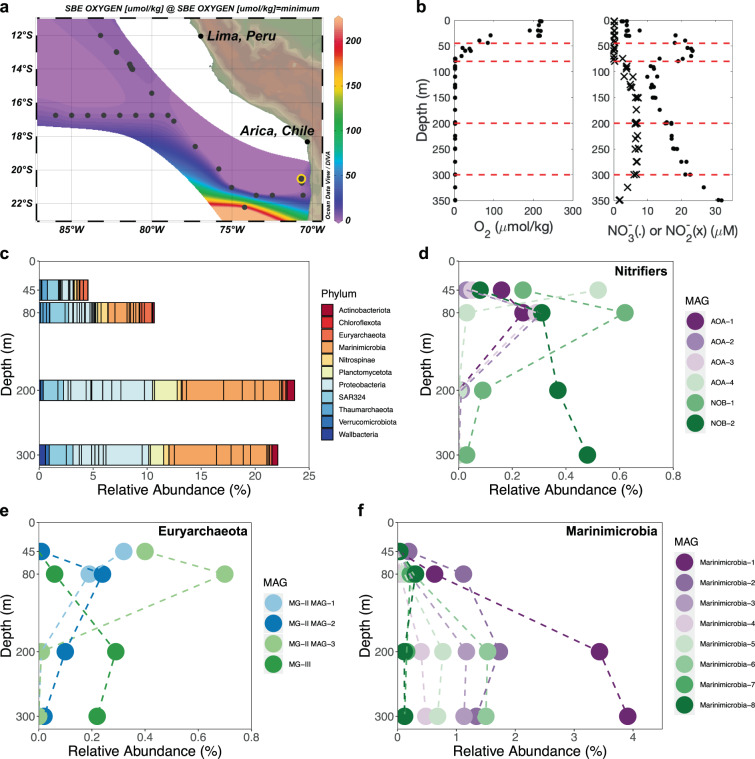
Sampling station information and relative abundances of MAGs. **a** Sampling station (yellow circle) in the ETSP OMZ and the minimum oxygen concentration at the time of the cruise. **b** Depth profiles of oxygen, nitrate, and nitrite reported previously [7] at the sampling station and the four sample depths indicated by red dashed lines (45, 80, 200, and 300 m). **c** Relative abundances of MAGs in the ETSP OMZ grouped by phylum. **d**–**f** The distribution of individual MAGs in four phyla discussed in the text. The relative abundance of NOB-1 and NOB-2 was reported previously [12].

**Fig. 2:**
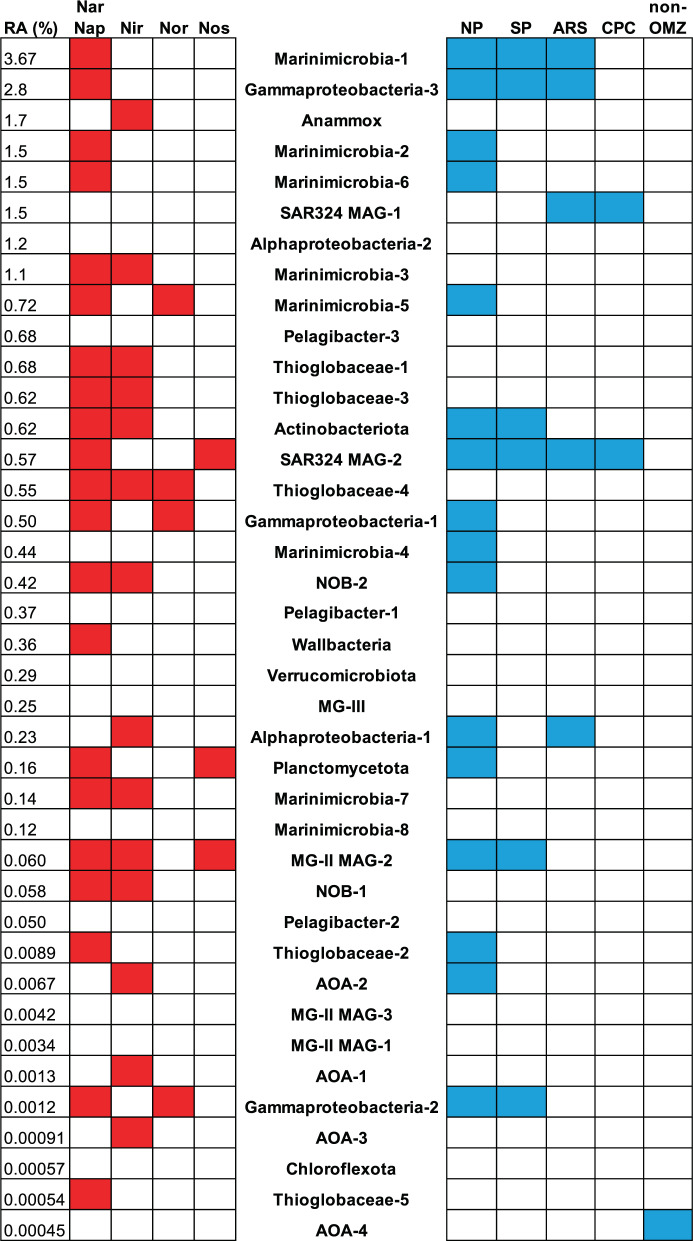
Preliminary prediction of nitrogen cycling metabolisms and representation of MAGs in global ocean. Presence (red) and absence (white) of genes involved in the denitrification pathway (Nar or Nap nitrate reductase, Nir nitrite reductase, Nor nitric oxide reductase, Nos N_2_O reductase) for each MAG. Relative abundance (RA) of MAGs in the anoxic core of the ETSP OMZ (averaged between 200 and 300 m). MAGs from this study are ordered by their relative abundance in the anoxic core. Presence (blue) and absence (white) of MAGs from Tara Oceans datasets, which were identified as the same species with MAGs from this study. Tara Oceans regions, where Tara Oceans MAGs were originally found, include four regions with OMZ sites: NP North Pacific region, SP South Pacific region, ARS Arabian Sea region, CPC Chile-Peru Coastal region, and non-OMZ regions including North Atlantic, South Atlantic, East Africa Coastal, Mediterranean, Red Sea, and Indian Ocean regions.

Oxygen minimum zones (OMZs) are unique oceanic regions with strong redox gradients. Anoxic zones in OMZs are hotspots for fixed nitrogen loss and production of the greenhouse gas N_2_O [1, 2]. Microbes in OMZs make important contributions to biogeochemistry, which motivates us to reconstruct metagenome-assembled genomes (MAGs) from the Eastern Tropical South Pacific (ETSP) OMZ (Fig. 1a, b). Among 147 recovered MAGs, we present 39 high- and medium-quality MAGs with completeness >50% and contamination <10% [3], including 8 archaeal and 31 bacterial MAGs (Fig. S1 and Table S1) representing 11 phyla (Fig. 1c). Methods of MAG construction and analysis are available in the supplement. We compared these new OMZ MAGs to 2631 MAGs recovered from the most comprehensive marine microbial metagenomic datasets (Tara Oceans) [4], which included OMZ and non-OMZ sites. More than half of these ETSP OMZ MAGs were not represented at the species level in the Tara Oceans dataset (Fig. 2 and Supplementary Methods). These 39 ETSP MAGs only represented up to 24% of the total microbial population (Fig. 1c), thus many more novel species in OMZs remain to be discovered. Seventeen ETSP OMZ MAGs were identified as the same species in regions, where OMZ sites were included in the Tara Oceans dataset (Fig. 2), indicating adaptation to the unique OMZ environment and the necessity to explore these OMZ MAGs for novel taxa and functional potentials.

Taxonomy-resolved MAGs recovered here will allow linking previously measured biogeochemical cycling rates on the same cruise [5–10] to their microbial drivers. The most abundant fixed nitrogen in the ocean, nitrate, is produced via nitrification. The first step of nitrification, ammonia oxidation, is mainly performed by marine ammonia-oxidizing archaea (AOA) [11], and then nitrite-oxidizing bacteria (NOB) oxidize nitrite into nitrate. Novel niches of NOB were discovered by analyzing the two NOB MAGs from this dataset [12]. Kinetics experiments at other OMZ stations suggested distinct oxygen affinities of AOA and NOB [13–15]. In anoxic waters, ammonia oxidation rates were undetectable, but nitrite oxidation rates were high (>100 nM d^−1^) at the same station [6], where MAGs were recovered. Consistently, Thaumarchaeota MAGs (AOAs) were nearly absent (only AOA-2 had a relative abundance higher than 0.01%) and NOB MAGs (NOB-1 and NOB-2) were much more abundant than AOA in the anoxic core (Fig. 1d). MAGs in this study will provide opportunities to discover novel processes and adaptation strategies.

Most MAGs had their highest relative abundances in the anoxic zone (Fig. 1c). Many of them contribute to the loss of fixed nitrogen, which occurs by denitrification (the sequential reduction of nitrate to nitrite, NO, N_2_O, and finally N_2_) and anammox (anaerobic oxidation of ammonium to N_2_). Measured nitrate reduction rates at this [5, 8] and other [16, 17] nearby stations were much larger than rates of any subsequent denitrification steps (e.g., nitrite reduction to N_2_O or to N_2_). Consistently, preliminary prediction of metabolisms shows that more than half of the MAGs found here contained *nar*, which encodes nitrate reduction, and one-third of those contained only *nar* and none of the other denitrification genes (i.e., they are nitrate-reducing specialists) (Fig. 2). Consistently, a previous study found that *nar* dramatically outnumbered the other denitrification genes in contigs from the Eastern Tropical North Pacific (ETNP) OMZ [18]. Indeed, four of the five most abundant MAGs in the anoxic core were nitrate-reducing specialists (Fig. 2). The fifth was an anammox MAG, which was only assigned to the genus level (*Candidatus* Scalindua) in GTDB and was not represented at the species level in the Tara Oceans dataset (Table S1). However, this anammox MAG was highly related to 20 anammox single-cell amplified genomes (SAGs) from the ETNP OMZ [19]. The anammox MAG had at least 90% average nucleotide identity (ANI) to the SAGs, with the highest ANI (98.8%) to SAG K21. Consistent with the previous work [19], the anammox MAG also encoded cyanase, indicating its potential of using organic nitrogen substrates. The most abundant nitrate reducer MAG here is Marinimicrobia-1 (Fig. 1), which belongs to the newly proposed phylum *Candidatus* Marinimicrobia [20]. Notably, one nitrate reducer can only be assigned to phylum level (*Candidatus* Wallbacteria) and was not present in the Tara Oceans MAGs (Table S1).

We also identified a novel archaeal MAG possessing multiple denitrification genes. MG-II MAG-2 encoded Nar alpha and beta subunits, nitrate/nitrite transporters, copper-containing nitrite reductase, and N_2_O reductase (Fig. 2). Two MAGs from the Tara Oceans metagenomes (Table S1) were identified as the same species as MG-II MAG-2. TOBG_NP-110 (ANI to MG-II MAG-2 = 99.8%) from the North Pacific encoded Nar and nitrate/nitrite transporters, and TOBG_SP-208 (ANI to MG-II MAG-2 = 99.6%) from the South Pacific also contained the same denitrification genes as MG-II MAG-2 (Table S2). In addition, two MG-II SAGs (AD-615-F09 and AD-613-O09) were found at a different station of the ETSP OMZ sampled on the same cruise as this study [21]. Partial 16S rRNA genes of both SAGs are 100% identical to that of MG-II MAG-2 (alignment length = 200 bp for AD-615-F09 and 183 bp for AD-613-O09), but only AD-615-F09 might be the same species as MG-II MAG-2 based on ANI analyses (MG-II MAG-2 had 99.5% ANI to AD-615-F09, and 80.9% to AD-613-O09). Both SAGs also encoded Nar and nitrate/nitrite transporters [21]. The absence of other denitrification genes may be due to the low completeness of the two SAGs (completeness = 5.61% for both SAGs) [21]. Nitrite reductase and N_2_O reductase genes were located on the same contig in both MG-II MAG-2 and TOBG_SP-208 (Table S2). MG-II MAG-2 and TOBG_SP-208 had low contamination (1.9% and 0.8%, respectively), and their contigs with nitrite reductase and N_2_O reductase genes contained single-copy marker genes present only once in each MAG (Supplementary Methods). Although these results suggest a nearly complete denitrification metabolism in MG-II archaea, especially N_2_O consumption metabolism, methods besides metagenomics (e.g. reconstructing SAGs with high completeness) are highly recommended to rule out possible artifacts introduced by metagenomic binning and confirm the presence of these genes and their denitrification activity. Nonetheless, MG-II MAG-2 was present (Fig. 1e) and transcriptionally active in both Pacific OMZs (Fig. S2), indicating its adaptation to low oxygen environments. The MG-III MAG did not have any denitrification genes but was abundant in the anoxic zone (Figs. 1e and 2). It had a GC value (43.2%) distinct from all other known MG-III MAGs [22] and is the most complete (86.0%) and the least contaminated (0%) (Table S1) among all reported MG-III MAGs, indicating that MG-III is a novel archaeon in this group. Bacterial and archaeal MAGs recovered here implied that nitrogen metabolisms were present in more microbial lineages than previously thought. Further analyses of these MAGs will shed light on adaptation strategies in the unique OMZ environment and novel functions related to important element cycles.

## Supplementary information

Supplementary information

Supplementary Table 1

Supplementary Table 2

## Data Availability

Raw metagenomic reads used to construct MAGs in this paper were submitted to NCBI with the accession numbers SRR14610252, SRR14610253, SRR14610254, and SRR14610255. MAGs analyzed in this paper were deposited at https://figshare.com/articles/MAGs_from_ETSP_OMZ/12291281.
