# Supplementary material for: Novel metagenome-assembled genomes involved in the nitrogen cycle from a Pacific oxygen minimum zone
Source: ISME Commun. 2021 Jun 18;1:26. doi: 10.1038/s43705-021-00030-2 (PMC9723717; doi:10.1038/s43705-021-00030-2)
Supplement: Supplementary file 1 — Supplementary information [file 43705_2021_30_MOESM1_ESM.pdf]

Supplementary information for

Novel metagenome-assembled genomes involved in the nitrogen cycle from a Pacific oxygen minimum zone

Xin Sun, Bess B. Ward

This document includes:

Methods

Figure S1 and Figure S2

The captions of Table S1 and Table S2

References

Methods

Sample collection, DNA extraction, and metagenomic sequencing were described in a previous study exploring two MAGs from this dataset [1]. Briefly, four particulate material samples were collected by filtering (0.2 μm pore size) seawater from the ETSP OMZ (20.50°S, 70.70°W, Fig. 1a) aboard the R/V Nathaniel B. Palmer (NBP 1305) in 2013. The four samples captured distinct features of the OMZ: the oxycline where oxygen is decreasing sharply (45 m), the oxic-anoxic interface (80 m), and the core of the anoxic zone with high nitrite concentrations (200 m and 300 m, Fig. 1b). All environmental variables such as temperature, oxygen and nutrient concentrations from this cruise are available at BCO-DMO (https://www.bco-dmo.org/dataset/744679). DNA was extracted from four filters and raw reads were generated from Illumina MiSeq sequencing. Quality control, co-assembly of four metagenomes, and binning processes were also described in detail in the previous study[1]. Briefly, raw reads were trimmed and filtered using BBDuk from BBTools package (available at: https://jgi.doe.gov/data-and-tools/bbtools/). Reads from four metagenomes were co-assembled into contigs using metaSPAdes [2]. Coverage information of contigs was obtained by mapping reads from four samples to contigs using Burrows-Wheeler Aligner [3] and SAMtools [4]. Contigs equal to or longer than 1500 bp were binned into MAGs using BinSanity [5], COCACOLA [6], CONCOCT [7], MaxBin [8], and MetaBAT [9]. Finally, DAS Tool was used to combine results from different binning methods to obtain final MAGs [10].

The quality of OMZ MAGs reconstructed in this study was assessed using checkM [11]. High- and Medium-quality (HQMQ) MAGs (39 MAGs) with completeness >50% and contamination <10% [12] were included in further assessments. The taxonomy of 39 OMZ MAGs was predicted using GTDB-tk v1.0.2 with database r89 [13]. Taxonomy of published MAGs from Tara Ocean datasets [14] was also predicted using GTDB-tk [13] to assess the taxonomy coverage of OMZ MAGs in the global ocean. Most OMZ MAGs were named based on GTDB taxonomy classification, except for AOA, NOB, and anammox. MAGs from Tara Ocean identified as the same species as the new ETSP OMZ MAGs were recognized mainly based on GTDB taxonomy, i.e., being assigned to the same species. When OMZ MAGs and Tara Ocean MAGs were found to be the same genus but could not be further assigned to the species level due to the lack of reference genomes in GTDB, average nucleotide identity (ANI) values were calculated using OrthoANI v1.2 [15] with the parameter '-fmt matrix' to assess whether these MAGs belong to the same species (species ANI ≥ 95%). Taxonomy classification based on OrthoANI and GTDB is presented in

Online-only Table 1. ANI values were also calculated for anammox MAG and 20 anammox SAGs from the ETNP OMZ [16], and for MG-II MAG-II and two SAGs from the ETSP OMZ [17].

Selected metabolic potentials of MAGs were predicted to identify MAGs with significant biogeochemical implications for future studies. First, protein-coding sequences were predicted using Prodigal v2.6.3 [18] with '-p meta' mode. Then, BLASTp (v2.2.29) searches (e-value cutoff: 10^{-10}) were performed against the NCBI nr database with parameters '-max_target_seqs 1, -outfmt 11' and Blast_formatter (-outfmt "6 qseqid qlen sseqid slen qstart qend sstart send length pident nident mismatch evalue bitscore staxids saccver stitle"). In particular, single-copy marker genes present once and multiple times in MG-II MAG-2 and TOBG_SP-208 were located using checkM [11]. Contigs with nitrite reductase and N_2O reductase genes in MG-II MAG-2 and TOBG_SP-208 contained one and four marker genes, respectively, and all these marker genes were present only once in each MAG, implying that the denitrification gene containing contigs might not be contaminants. However, further metabolic potential predictions and validation of these preliminary predictions are strongly recommended for future studies on these MAGs. Relative abundances of MAGs at the four different depths of the ETSP OMZ station were estimated by mapping reads from each depth using Bowtie2 v2.2.5 [19] with the parameter '--very-sensitive', and only reads with a mapping quality above 20 were included as mapped reads using SAMtools v1.5 view [4] with the parameter '-q 20'. The relative abundance of a MAG = (reads mapped to the MAG from a depth)/(total reads from this depth). Relative abundances of selected MAGs are visualized in Fig. 1, and relative abundances of all 39 MAGs are presented in Online-only Table 1. Using similar approaches, metagenomes on different particle fractions from ETSP OMZ collected on other cruises [20] could be mapped to OMZ MAGs reconstructed here to link microbial taxonomy, functional potentials, and habitats.

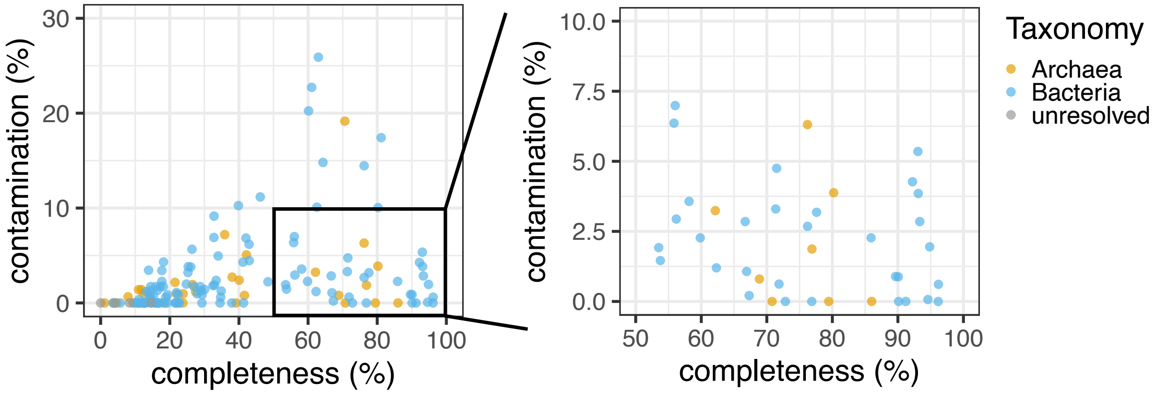

Fig. S1 Completeness and contamination of 147 MAGs from the ETSP OMZ, including 39 HQMQ MAGs.

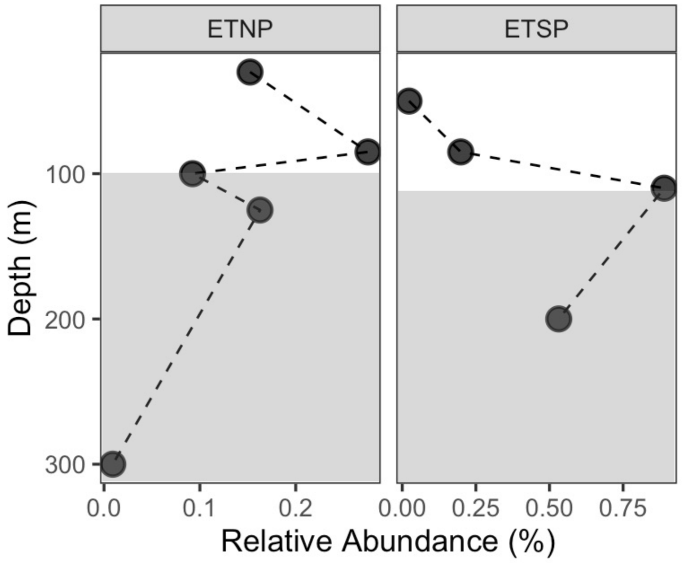

Fig. S2 Transcriptional activity of MG-II MAG-2 in ETNP and ETSP OMZs. Previously published metatranscriptomic reads from ETNP [21] and ETSP [22] were mapped to MG-II MAG-2. Anoxic zones are indicated by grey shaded areas.

Table S1 Genome characteristics and relative abundance of MAGs (additional excel file attached).

Table S2 Annotation of contigs containing denitrification genes in selected MAGs (additional excel file attached).

References

1. Sun X, Kop LFM, Lau MCY, Frank J, Jayakumar A, Lücker S, et al. Uncultured Nitrospina-like species are major nitrite oxidizing bacteria in oxygen minimum zones. *ISME J* 2019; **13**: 2391–2402.
2. Nurk S, Meleshko D, Korobeynikov A PP. metaSPAdes: A New Versatile Metagenomic

Assembler. *Genome Res* 2017; **1**: 30–47.
3. Li H, Durbin R. Fast and accurate short read alignment with Burrows-Wheeler transform. *Bioinformatics* 2009; **25**: 1754–1760.
4. Li H, Handsaker B, Wysoker A, Fennell T, Ruan J, Homer N, et al. The Sequence Alignment/Map format and SAMtools. *Bioinformatics* 2009; **25**: 2078–2079.
5. Graham ED, Heidelberg JF, Tully BJ. Binsanity: Unsupervised clustering of environmental microbial assemblies using coverage and affinity propagation. *PeerJ* 2017; **2017**: 1–19.
6. Lu YY, Chen T, Fuhrman JA, Sun F, Sahinalp C. COCACOLA: Binning metagenomic contigs using sequence COmposition, read CoverAge, CO-alignment and paired-end read LinkAge. *Bioinformatics* 2017; **33**: 791–798.
7. Alneberg J, Bjarnason BS, De Bruijn I, Schirmer M, Quick J, Ijaz UZ, et al. Binning metagenomic contigs by coverage and composition. *Nat Methods* 2014; **11**: 1144–1146.
8. Wu Y-W, Simmons BA, Singer SW. MaxBin 2.0: an automated binning algorithm to recover genomes from multiple metagenomic datasets. *Bioinformatics* 2016; **32**: 605–607.
9. Kang DD, Froula J, Egan R, Wang Z. MetaBAT, an efficient tool for accurately reconstructing single genomes from complex microbial communities. *PeerJ* 2015; **3**: e1165.
10. Sieber CMK, Probst AJ, Sharrar A, Thomas BC, Hess M, Tringe SG, et al. Recovery of genomes from metagenomes via a dereplication, aggregation and scoring strategy. *Nat Microbiol* 2018; **3**: 836–843.
11. Parks DH, Imelfort M, Skennerton CT, Hugenholtz P, Tyson GW. CheckM: assessing the quality of microbial genomes recovered from isolates, single cells, and metagenomes. *Genome Res* 2015; **25**: 1043–55.
12. Bowers RM, Kyrpides NC, Stepanauskas R, Harmon-Smith M, Doud D, Reddy TBK, et al. Minimum information about a single amplified genome (MISAG) and a metagenome-assembled genome (MIMAG) of bacteria and archaea. *Nat Biotechnol* 2017; **35**: 725–731.
13. Chaumeil P-A, Mussig AJ, Hugenholtz P, Parks DH. GTDB-Tk: a toolkit to classify genomes with the Genome Taxonomy Database. *Bioinformatics* 2019; **36**: 1925–1927.
14. Tully BJ, Graham ED, Heidelberg JF. The reconstruction of 2,631 draft metagenome-assembled genomes from the global oceans. *Sci Data* 2018; **5**: 170203.
15. Lee I, Ouk Kim Y, Park S-C, Chun J. OrthoANI: An improved algorithm and software for calculating average nucleotide identity. *Int J Syst Evol Microbiol* 2016; **66**: 1100–1103.
16. Ganesh S, Bertagnolli AD, Bristow LA, Padilla CC, Blackwood N, Aldunate M, et al. Single cell genomic and transcriptomic evidence for the use of alternative nitrogen substrates by anammox bacteria. *ISME J* 2018; **12**: 2706–2722.
17. Plominsky AM, Trefault N, Podell S, Blanton JM, De la Iglesia R, Allen EE, et al. Metabolic potential and in situ transcriptomic profiles of previously uncharacterized key microbial groups involved in coupled carbon, nitrogen and sulfur cycling in anoxic marine zones. *Environ Microbiol* 2018; **20**: 2727–2742.
18. Hyatt D, Chen G, Locascio PF, Land ML, Larimer FW, Hauser LJ. Prodigal : prokaryotic gene recognition and translation initiation site identification. *BMC Bioinformatics* 2010; **11**: 1–11.
19. Langmead B, Salzberg SL. Fast gapped-read alignment with Bowtie 2. *Nat Methods* 2012; **9**: 357–360.
20. Ganesh S, Parris DJ, DeLong EF, Stewart FJ. Metagenomic analysis of size-fractionated

picoplankton in a marine oxygen minimum zone. *ISME J* 2014; **8**: 187–211.
21. Ganesh S, Bristow LA, Larsen M, Sarode N, Thamdrup B, Stewart FJ. Size-fraction partitioning of community gene transcription and nitrogen metabolism in a marine oxygen minimum zone. *ISME J* 2015; **9**: 2682–2696.
22. Stewart FJ, Ulloa O, DeLong EF. Microbial metatranscriptomics in a permanent marine oxygen minimum zone. *Environ Microbiol* 2012; **14**: 23–40.
